# Functionally Orthologous Viral and Cellular MicroRNAs Studied by a Novel Dual-Fluorescent Reporter System

**DOI:** 10.1371/journal.pone.0036157

**Published:** 2012-04-27

**Authors:** Xiangyu You, Zhiping Zhang, Jinyu Fan, Zongqiang Cui, Xian-En Zhang

**Affiliations:** 1 State Key Laboratory of Virology, Wuhan Institute of Virology, Chinese Academy of Sciences, Wuhan, China; 2 Graduate School, Chinese Academy Sciences, Beijing, China; National Institutes of Health, United States of America

## Abstract

Recent research raised the possibility that some viral microRNAs (miRNAs) may function as orthologs of cellular miRNAs. In the present work, to study the functional orthologous relationships of viral and cellular miRNAs, we first constructed a dual-fluorescent protein reporter vector system for the easy determination of miRNA function. By expressing the miRNAs and the indicator and internal control fluorescent proteins individually from a single vector, this simple reporter system can be used for miRNA functional assays that include visualizing miRNA activity in live cells. Sequence alignments indicated that the simian virus 40 (SV40) encoded miRNA sv40-mir-S1-5p contains a seed region identical to that of the human miRNA hsa-miR423-5p. Using the new reporter system, it was found that sv40-mir-S1-5p and hsa-miR423-5p downregulate the expression of common artificial target mRNAs and some predicted biological targets of hsa-miR423-5p, demonstrating that they are functional orthologs. The human immunodeficiency virus 1 (HIV-1) encoded hiv1-miR-N367 also contains a seed sequence identical to that of the human miRNA hsa-miR192. Functional assays showed that hiv1-miR-N367 and hsa-miR192 could downregulate common artificial and predicted biological targets, suggesting that these miRNAs may also act as functional orthologs. Thus, this study presents a simple and universal system for testing miRNA function and identifies two new pairs of functional orthologs, sv40-mir-S1-5p and hsa-miR423-5p as well as hiv-1-miR-N367 and hsa-miR192. These findings also expand upon our current knowledge of functional homology and imply that a more general phenomenon of orthologous relationships exists between viral and cellular miRNAs.

## Introduction

MicroRNAs (miRNAs) are a class of small regulatory RNAs (approximately 18 to 24 nucleotides in length) that are expressed in eukaryotes and viruses ([1], [2], [3]; reviewed by [4], [5]). miRNAs play vital roles in post-transcriptional regulation of gene expression, thus participating in multiple cellular biological processes or in the viral replication cycle [4], [5]. In animal cells, cellular miRNAs repress the expression of specific mRNAs by base pairing by the miRNA “seed region” (usually from the 2^nd^ to 8^th^ nucleotides) to the 3′-untranslated region (3′-UTR) of target mRNAs [4]. Viral miRNAs could repress not only host cellular genes but also viral genes to benefit viral replication by employing both miRNA and siRNA (small interfering RNA) pathways [6], [7], [8]. Interestingly, several recent publications have raised the new idea that viral miRNAs act as functional orthologs of cellular miRNAs [9], [10], [11]. These orthologs may play complex roles in virus-host interactions. However, until now, only one cellular miRNA, miR155, has been verified to be a functional ortholog of some viral miRNAs [9], [10], [11]. It remains uncertain whether there are other cellular and viral miRNAs orthologous pairs, which would imply that this represents a more general phenomenon.

Determining miRNA function is not an easy task. Some methods have been designed previously to detect miRNA functions [12], [13], [14], [15], [16]. The most widely used system is based on the luciferase reporter genes whose 3′-UTRs are connected to the target sequence of the miRNAs. In a typical miRNA functional assay using the luciferase reporter system, the levels of miRNA inhibition of the reporter genes were determined with both a miRNA producer construct and a Renilla firefly luciferase indicator control construct [16]. In this system, miRNA inhibition decreases the reporter signal. It is difficult to coordinately control the expression of the miRNAs, reporters and controls because of the co-transfection of multiple plasmids. More importantly, the cellular luciferase signal needs to be blocked to enable the observation of dynamic miRNA activity. Recently, lentiviral or retroviral vectors encoding fluorescent proteins connected to a target sequence were also constructed for miRNA functional studies [17], [18]. These systems represent powerful tools for monitoring the dynamic function of miRNAs in single living cells. However, the present fluorescent protein reporter systems are designed uniquely for monitoring a specific miRNA. In addition, these systems require the production and integration of lentivirus. These features make the present reporter systems inconvenient and inflexible with regards to the study of multiple miRNA functions.

In the present work, to study the functional orthologous relationship of viral and cellular miRNAs, we first constructed a simple dual-fluorescent protein reporter system to assay miRNA function. Our strategy is based on placing the encoded reporter protein, control fluorescent proteins and miRNAs into a single vector with each component being expressed under the control of one of three separate promoters. The reporter system was successfully used to determine miRNA function and to visualize the action of miRNAs in live cells by microscopy. To find new virus-encoded miRNAs that may act as orthologs of cellular miRNAs, we examined the miRNAs encoded by the DNA virus Simian Virus 40 (SV40) and the RNA virus Human Immunodeficiency Virus-1 (HIV–1) for any sequence homologies with human-derived miRNAs listed in miRBase (http://microrna.sanger.ac.uk/). One of the SV40-encoded miRNAs, sv40-miR-S1-5p [7], was found to contain a seed sequence identical to that of hsa-miR423-5p. In addition, one of the HIV-1-encoded miRNAs, hiv1-miR-N367 [6], [8] (also called nef microRNA), contained a seed sequence identical to that of hsa-miR192. The seed sequence homologies suggested that these viral miRNAs may represent functional orthologs of cellular miRNAs [9], [10], [11]. With our newly constructed dual-fluorescent protein reporter system, we demonstrated that sv40-miR-S1-5p may function as an ortholog of cellular hsa-miR423-5p and that hiv1-miR-N367 may function as an ortholog of cellular hsa-miR192. This study provides a new tool to assay miRNA function and identifies two new functionally orthologous pairs of viral and cellular miRNAs.

## Results

### Design and construction of the dual-fluorescent protein reporter vector

**Figure 1:**
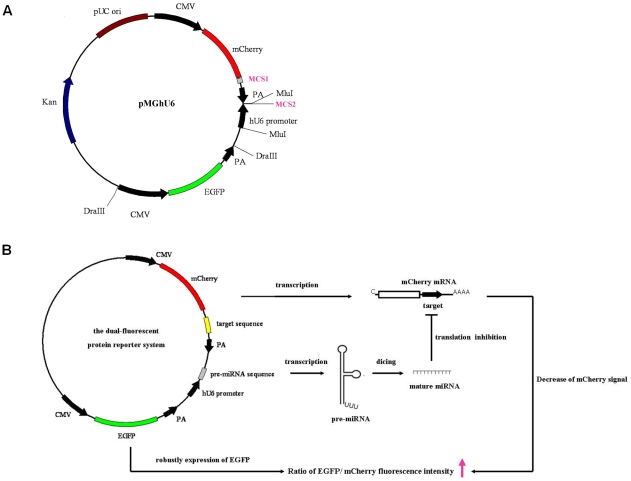
The construction and principle behind the dual-fluorescent protein reporter vector. (A) A diagram of the dual-fluorescent protein reporter vector pMGhU6. (B) The principle of the dual-fluorescent protein reporter vector system.

A dual-fluorescent protein reporter vector was constructed to assay miRNA function. As shown in Figure 1, the commercial vector pEGFP-C1 (Clontech) was reengineered to transcribe pre-miRNAs under the control of the human U6 promoter [19] and to separately express EGFP and mCherry under the control of their respective cytomegalovirus (CMV) immediate-early promoters. The resulting vector was named pMGhU6 (see Figure 1A). Multiple cloning site 1 (MCS1) (see Figure S1) in pMGhU6 allows for the insertion of miRNA target sequences into the 3′-UTR of the mCherry gene. Multiple cloning site 2 (MCS2), which contains the EcoR V and Not I sites that closely follow the human U6 promoter and were introduced during the cloning process, allow for the insertion of specific pre-miRNA sequences. pMGhU6 also contains an independent EGFP expression cassette to robustly translate EGFP as an internal control. The major principles by which the vector system works are shown in (see Figure 1B). Because miRNAs repress protein translation, the target sequence of the miRNA that is inserted down-stream of the mCherry gene (in MCS1) would decrease the mCherry signal in the presence of the corresponding miRNA. Therefore, the ratio of EGFP to mCherry fluorescence intensity is expected to increase compared to the control indicator vector that lacks any sites or contains unrelated target sites as well as the control cell lines that do not express the relevant miRNAs. By utilizing this new strategy and vector, we can also monitor the expression of miRNAs in living cells.

### Validation of the dual-fluorescent protein reporter system as an miRNA functional assay

**Figure 2:**
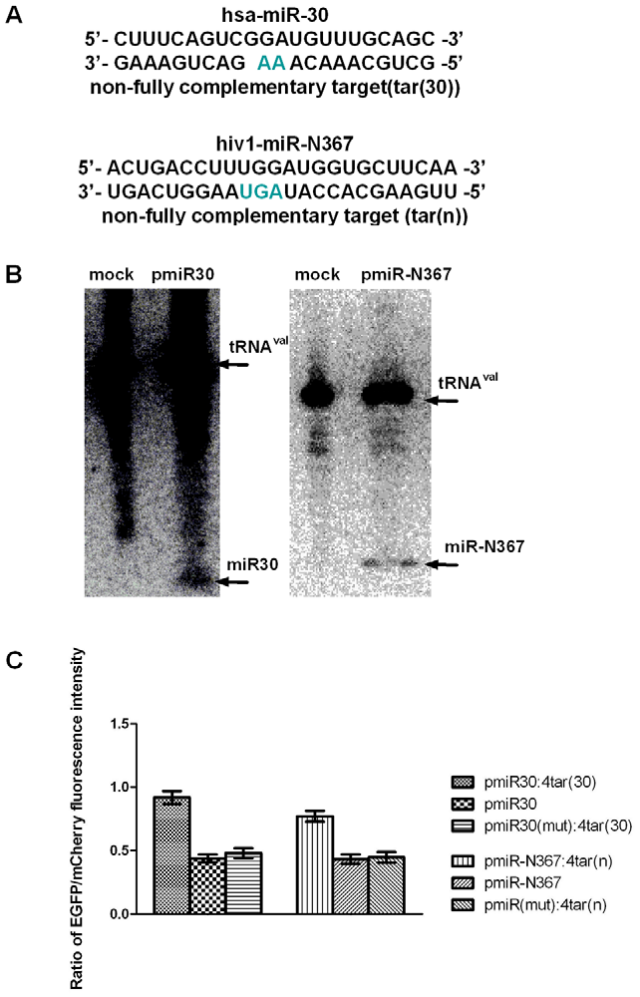
Validation of the dual-fluorescent protein reporter system as an miRNA functional assay. (A) Sequences of miR30, hiv1-miR-N367 and their non-fully complementary targets. (B) Northern blot analysis of the transcription of specific miRNAs. HeLa cells were mock-transfected (mock) or transfected with plasmids pmiR30, pmiR-N367 individually, and the location of the mature miR30 and miR-N367 is indicated. tRNA^Val^ served as a loading control [26]. (C) Fluorescence reporter assay of the indicator vectors pmiR30:4tar(30), pmiR-N367:4tar(n) and their control vectors. HeLa cell cultures were transfected with the indicator vectors pmiR30:4tar(30), pmiR-N367:4tar(n) and their control vectors with miRNAs or target sequences only. The ratio of EGFP to mCherry fluorescence intensity is shown.

To assess the efficacy of the dual-fluorescent protein reporter vector, we constructed a series of vectors producing different miRNAs that are derived from different species based on pMGhU6. The vectors pmiR30 and pmiR-N367 were constructed to produce high levels of the human-encoded miR30 [12] and the HIV-1-encoded miR-N367, respectively. Based on the above vectors, we constructed the indicator vectors pmiR30:4tar(30) and pmiR-N367:4tar(n), which contain tandem copies of the different targets (see Figure 2A) to specific miRNAs in the 3′-UTR of the mCherry gene and their negative control vectors, which do not produce targets or miRNAs, by deleting the miRNA-encoding region of each vector.

The expression of the vector-derived miRNAs was first examined for the dual-fluorescent protein reporter vectors. As shown in Figure 2B, Northern blot analysis readily revealed detectable levels of the specific miRNAs in all of the HeLa cultures transfected with constructs producing the pre-miRNA sequences of miR30 and miR-N367. These miRNAs were undetectable in HeLa cells that were not transfected. The experission of miRNAs from transfected vectors were also verified by real-time quantitative PCR (see Figure S4).

To perform the fluorescence reporter assay, HeLa cells were transfected with the indicator vectors pmiR30:4tar(30), pmiR-N367:4tar(n) and their control vectors, which did not produce target sequences or relevant miRNAs, respectively. Following transfection of the individual vectors (0.4 μg DNA per well in a 24-well plate), the fluorescence intensity of EGFP and mCherry was measured at 48 hours post-transfection using a Synergy HT instrument with an excitation wavelength of 485±20 nm and an emission wavelength of 528±20 nm for the EGFP channel and an excitation wavelength of 590±20 nm and an emission wavelength of 645±40 nm for the mCherry channel. The ratio of EGFP to mCherry fluorescence intensity was then determined. The results from the fluorescence reporter assay revealed that the ratio of EGFP to mCherry fluorescence intensity of cells with pmiR30:4tar(30) and pmiR-N367:4tar(n) was significantly elevated compared to cells with the control vectors that did not contain target sites and did not produce any relevant miRNAs (see Figure 2C). The results further showed that miRNAs derived from the vectors repressed the translation of mCherry proteins whose 3′-UTR contained tandem copies of the targets of the specific miRNA. And the results were consistent when diferent amounts of vectors were used (Figure S5). Therefore, this new reporter system successfully determines specific miRNA activity. The system is simple and reliable because it is based on the combination of the miRNA producer, indicator and internal control in a single construct.

### Monitoring miRNA activity in single living cells

**Figure 3:**
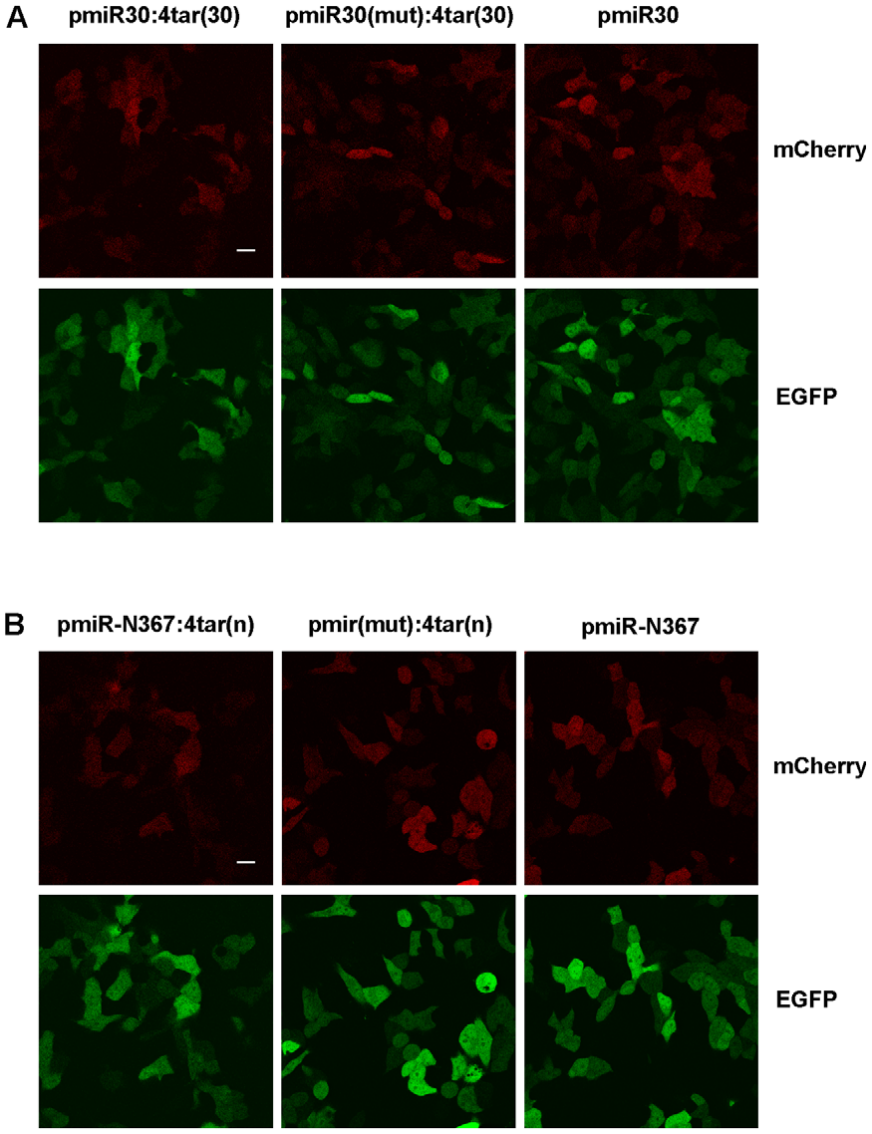
Monitoring miRNA activities in living cells. Activities of two miRNAs, including miR30 (A), miR-N367 (B), were imaged in live HeLa cell cultures that were transfected with the indicator vectors pmiR30:4tar(30), pmiR-N367:4tar(n) and their control vectors with miRNAs or target sequences only, respectively. Bar, 10 μm.

The dual-fluorescent protein report system was also used to image the activity of miRNAs in living cells. HeLa cells were transfected with the indicator vectors pmiR30:4tar(30), pmiR-N367:4tar(n) and their control vectors, which do not produce target sequences or relevant miRNAs. At 48 hours post-transfection, the fluorescence signals of both mCherry and GFP in HeLa cells were imaged using fluorescence microscopy. When all of the images were captured using the same exposure conditions, we could observe a reduction in mCherry expression in the HeLa cells with the indicator vectors pmiR30:4tar(30) and pmiR-N367:4tar(n) compared to the HeLa cells with the negative control vectors that did not produce target sequences (see Figure 3). The suppressive effect was specific for the mCherry that was connected to the target sequence because the internal control GFP fluorescence signals were not reduced in HeLa cells containing the vectors that did not produce target sequences. Similarly, mCherry signals from cells with pmiR30:4tar(30) and pmiR-N367:4tar(n) were clearly decreased compared to cells with vectors that produced target sequences but no miRNAs (see Figure 3). These results are consistent with the previous data using the fluorescence reporter assay and suggest that the dual-fluorescent protein report vector is also a good system to monitor the activity of miRNAs in living cells.

### sv40-miR-S1-5p and hsa-miR423-5p contain the same seed sequence and target common artificial mRNA sequences

**Figure 4:**
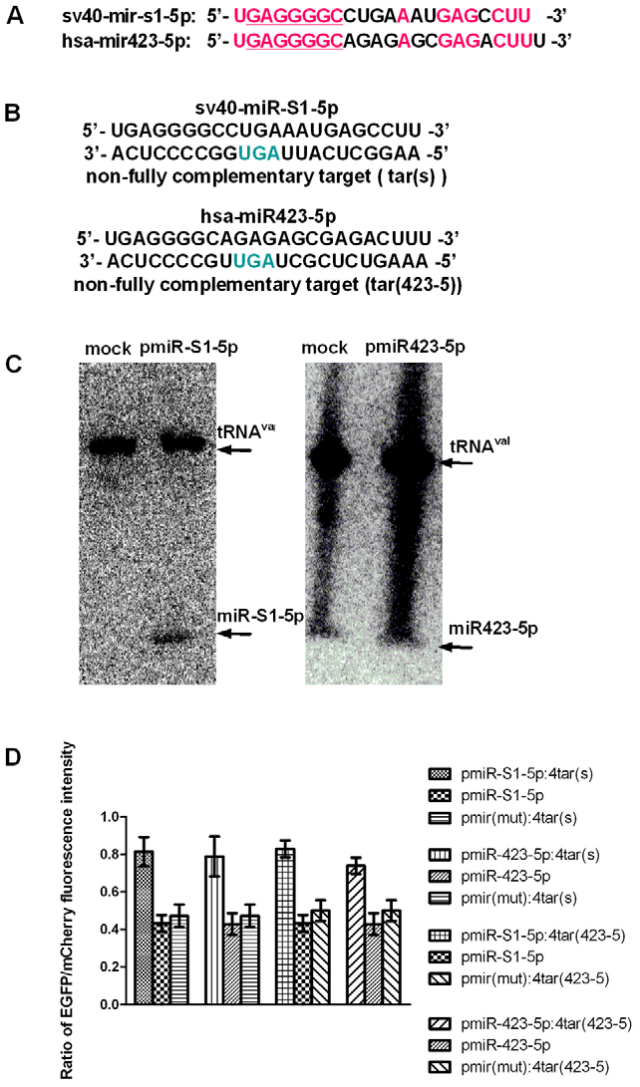
sv40-miR-S1-5p and hsa-miR423-5p share a seed sequence and target common artificial mRNA sequences. (A) Sequence homology between sv40-miR-S1-5p and hsa-miR423-5p (marked in red). The seed regions of miRNAs were underlined. (B) Sequences of sv40-miR-S1-5p, miR423-5p and their non-fully complementary targets. (C) Northern blot analysis of the transcription of miR-S1-5p and miR423-5p. HeLa cells were mock-transfected (mock) or transfected with plasmids pmiR-S1-5p and pmiR423-5p individually. The location of the mature miR-S1-5p and miR423-5p is indicated. tRNA^Val^ served as a loading control. (D) Fluorescence reporter assay of the indicator vectors pmiR-S1-5p:4tar(s), pmiR423-5p:4tar(s), pmiR-S1-5p:4tar(423-5), pmiR423-5p:4tar(423-5) and their control vectors with miRNAs or target sequences only.

Recently, several groups have reported that virus-encoded miRNAs can modulate some of the target genes that are repressed by cellular miR155, thereby acting as functional orthologs of cellular miR155 [9], [10], [11]. To find new virus-encoded miRNAs that may act as orthologs of cellular miRNAs, we examined the miRNAs encoded by the DNA virus SV40 for any sequence homologies with human-derived miRNAs listed in miRBase (http://microrna.sanger.ac.uk/). One of the SV40-encoded miRNAs, sv40-miR-S1-5p [7], was found to contain a seed sequence that is identical to that of the human-derived miRNA, hsa-miR423-5p, demonstrating its potential as a functional ortholog of hsa-miR423-5p (see Figure 4A).

The vectors pmiR-S1-5p and pmiR423-5p, based on the above dual-fluorescent reporter vector, were constructed to strongly express miR-S1-5p and miR423-5p, respectively. As the native stem-loops of precursor miR423-5p and miR-S1-5p not only express the mature sequences of miR423-5p and miR-S1-5p, respectively, but also express miR423-3p and miR-S1-3p (http://microrna.sanger.ac.uk/) as byproducts, miR30-based precursor stem-loops were used to exclusively express miR423-5p and miR-S1-5p [9] (see Figure S2A, B). Based on these miRNA expression vectors, a series of indicator vectors that share a common set of artificial non-fully complementary target sequences were constructed. The indicator vectors pmiR-S1-5p:4tar(s) and pmiR423-5p:4tar(s) share a common set of four tandem copies of predicted target sequences (tar(s)). The indicators pmiR-S1-5p:4tar(423-5) and pmiR423-5p:4tar(423-5) share a common set of four tandem copies of predicted target sequences (tar(423-5)). The tar(s) sequence is a non-fully complementary target of miR-S1-5p, and the tar(423-5) sequence is a non-fully complementary target of miR423-5p (see Figure 4B). Northern blot analysis was first carried out to demonstrate that the miRNA were expressed from these vectors (see Figure 4C). The experission of miRNAs from transfected vectors were also verified by real-time quantitative PCR (see Figure S4). Vectors that did not produce relevant miRNAs or target sites were also constructed for use as negative controls. The results from the fluorescence reporter assays revealed that the ratios of EGFP to mCherry fluorescence intensity of cells with pmiR-S1-5p:4tar(s), pmiR423-5p:4tar(s), pmiR-S1-5p:4tar(423-5), and pmiR423-5p:4tar(423-5) were obviously elevated as compared to cells with control vectors that did not contain target sites or produce relevant miRNAs (see Figure 4D). These assays demonstrated that miR-S1-5p and miR423-5p can inhibit the expression of indicator vectors that contain both their own and each other's non-fully complementary target sequences.

### sv40-miR-S1-5p and hsa-miR423-5p downregulate the predicted biological targets of hsa-miR423-5p

**Figure 5:**
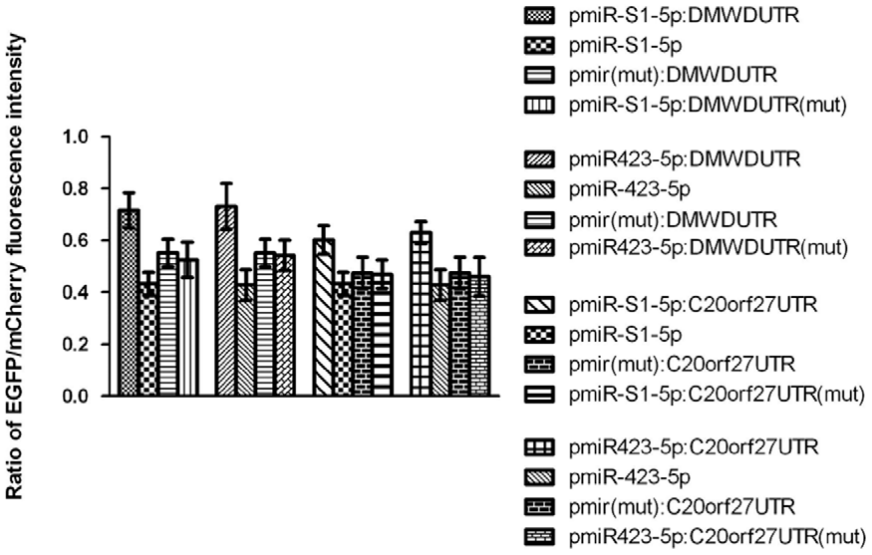
sv40-miR-S1-5p and hsa-miR423-5p downregulate the predicted biological targets of hsa-miR423-5p. HeLa cultures were transfected by the indicator vectors pmiR-S1-5p:DMWDUTR, pmiR423-5p:DMWDUTR, pmiR-S1-5p:C20orf27UTR, pmiR423-5p:C20orf27UTR and their control vectors with miRNAs only, with miRNA deletions or without miRNA pairing sites in the 3′-UTR.

To further test if miR-S1-5p and miR423-5p are functional orthologs of cellular miRNAs, we asked whether these miRNAs downregulate their predicted biological targets. Because it was difficult to find any predicted targets of sv40-miR-S1-5p, some predicted genes of miR423-5p were tested. The 3′-UTRs of the human genes DMWD and C20orf27, which contain several predicted “seed region” pairing sites of miR423-5p (See Figure S3A, B), were connected to the 3′-UTR of the mCherry gene in vectors pmiR-S1-5p and pmiR423-5p, respectively (http://www.targetscan.org/vert_50/). The indicator vectors pmiR-S1-5p:DMWDUTR, pmiR-S1-5p:C20orf27UTR, pmiR423-5p:DMWDUTR and pmiR423-5p:C20orf27UTR that are based on the miRNA expression vectors pmiR-S1-5p and pmiR423-5p were constructed for fluorescence reporter assays. The negative control vectors that do not produce relevant miRNAs were constructed by deleting the pre-miRNA-encoding region, and the negative control vectors that do not contain relevant miRNA binding sites within the relevant 3′-UTR were constructed by deleting the relevant miRNA binding sites using overlapping PCR. The results from the fluorescence reporter assays revealed that the ratios of EGFP to mCherry fluorescence intensity of cells with pmiR-S1-5p:DMWDUTR, pmiR-S1-5p:C20orf27UTR, pmiR423-5p:DMWDUTR and pmiR423-5p:C20orf27UTR were significantly elevated as compared to cells with control vectors that lack target sites and do not produce relevant miRNAs (see Figure 5). Thus, the reporter assays demonstrated that targets, such as DMWD and C20orf27, can be negatively regulated by both miR423–5p and miR–S1–5p. Together with the above results, these data demonstrate that the SV40-encoded miRNA miR-S1-5p may act as a functional ortholog of the human derived-miRNA miR423-5p.

### hiv1-miR-N367 and hsa-miR192 downregulate common artificial and predicted biological targets as functional orthologs

**Figure 6:**
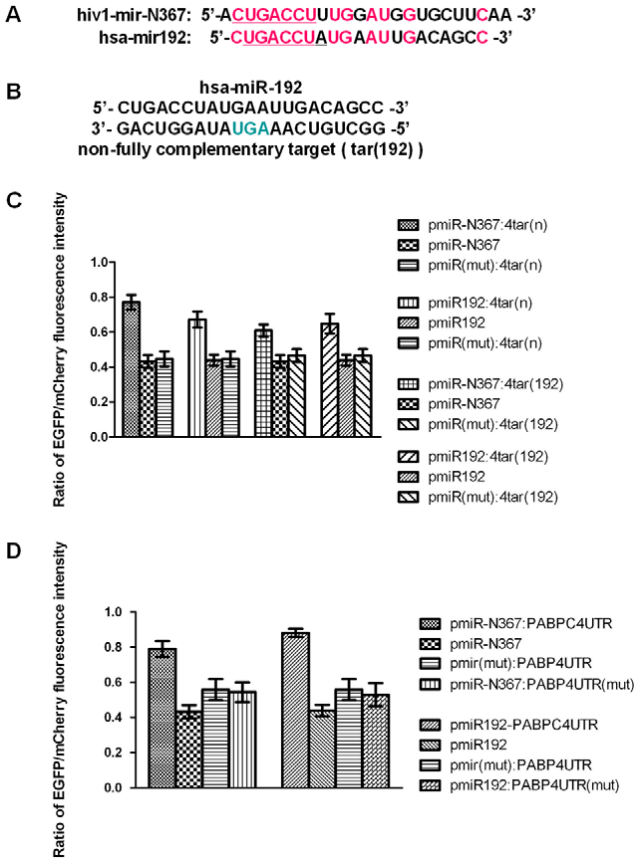
hiv1-miR-N367 and hsa-miR192 act as functional orthologs. (A) Sequence homology between hiv1-miR-N367 and hsa-miR192 (marked in red). The seed regions of miRNAs were underlined. (B) Sequences of miR192 and its non-fully complementary target. (C) Fluorescence reporter assay of the indicator vectors pmiR-N367:4tar(n), pmiR192:4tar(n), pmiR-N367:4tar(192), pmiR192:4tar(192) and their control vectors with miRNAs or target sequences only. (D) Fluorescence reporter assay of the indicator vectors pmiR-N367:PABPC4UTR, pmiR192:PABPC4UTR and their control vectors with miRNAs only, with miRNA deletions or without miRNA pairing sites in the 3′-UTR.

We also examined the miRNAs encoded by the RNA virus HIV-1 for any sequence homologies with human-derived miRNAs that are listed in miRBase (http://microrna.sanger.ac.uk/). One of the HIV-1-encoded miRNAs, hiv1-miR-N367 [6], [8], contains a seed sequence identical to that of hsa-miR192 (from 2^nd^ to 7^th^ nucleotides), demonstrating its potential as a functional ortholog of hsa-miR192 (see Figure 6A).

The construction of the vector pmiR-N367 that expresses miR-N367 is described above. The vector pmiR192 was constructed to express miR192 at high levels and is based on our dual-fluorescent reporter vector. As the native stem–loops of precursor miR192 not only express the mature sequence of miR192 but also express miR192* (http://microrna.sanger.ac.uk/) as a byproduct, the miR30-based precursor stem-loops were used to exclusively express miR192 [9] (see Figure S2C). Based on these miRNA expression vectors, a series of indicator vectors that share a common set of artificial non-fully complementary target sequences were constructed. The indicator vectors pmiR-N367:4tar(n), pmiR192:4tar(n), pmiR-N367:4tar(192) and pmiR192:4tar(192), which share a common set of four tandem copies of the predicted target sequences tar(n) and tar(192), were constructed. The tar(n) sequence is a non-fully complementary target of miR-N367, and the tar(192) sequence is a non-fully complementary target of miR192 (see Figure 6B). Northern blot and real-time quantitative PCR analysis were carried out to determine the miRNA expression from these vectors (data not shown). Vectors that did not produce relevant miRNAs or target sites were also constructed to serve as negative controls. The results from fluorescence reporter assays revealed that the ratios of EGFP to mCherry fluorescence intensity of cells with pmiR-N367:4tar(n), pmiR192:4tar(n), pmiR-N367:4tar(192) and pmiR192:4tar(192) were obviously elevated as compared to cells with control vectors that did not contain target sites or produce relevant miRNAs (see Figure 6C). These assays demonstrated that miR-N367 and miR192 can inhibit the expression of indicator vectors that contain their own and each other's non-fully complementary target sequences.

Additionally, some predicted biological targets were used to further test the orthologous functions of hiv1-miR-N367 and hsa-miR192. In this case, the 3′-UTR sequence of the human gene PABPC4 (see Figure S3C) was connected to the 3′-UTR of the mCherry reporter gene in the vectors pmiR-N367 and pmiR192. The 3′-UTR of the human gene PABPC4 contains two conserved sequential “seed region” pairing sites, which are predicted targets of miR192, as determined by TARGETSCAN and other miRNA target prediction algorithms (http://www.targetscan.org/vert_50/). The negative control vectors that did not produce relevant miRNAs were constructed by deleting the pre-miRNA-encoding region, and the negative control vectors that did not contain relevant miRNA binding sites within the relevant 3′-UTRs were constructed by deleting the relevant miRNA-binding sites using overlapping PCR. The results from the fluorescence reporter assays revealed that the ratios of EGFP to mCherry fluorescence intensity of cells with pmiR-N367:PABPC4UTR and pmiR192:PABPC4UTR were obviously elevated as compared to cells with control vectors that did not contain target sites or produce relevant miRNAs (see Figure 6D). Thus, the reporter assays demonstrate that targets, such as PABPC4, can be negatively regulated by both miR192 and miR-N367. These results further demonstrate that hiv1-miR-N367, an HIV-1-encoded miRNA, should act as a functional ortholog of the human-derived miRNA hsa-miR192.

## Discussion

Recently, it was found that virally encoded miR-K12-11 and miR-M4 from two oncogenic herpesviruses are functional orthologs of cellular miR155, an miRNA that performs multiple functions and could play a major role in lymphoid malignancies and the modulation of immune responses [9], [10], [11]. However, this observation might not be an anomaly. The seed region of the mature miRNA is critical for mRNA target recognition. Alignment of miRNAs of some viruses with human-derived miRNAs revealed that some additional viral miRNAs share seed homology to cellular miRNAs, which suggests the possibility that they might also be functionally orthologous miRNA pairs.

To easily detect the function of miRNAs, we first established a dual-fluorescent protein vector system to report on miRNA activity. In the vector pMGhU6 (see Figure 1), miRNAs are produced under the control of the human U6 promoter, and EGFP and mCherry are independently expressed under the control of two divergent CMV immediate-early promoters. miRNA target sequences can be placed into the 3′-UTR of the mCherry gene, and miRNA activity decreases the mCherry signal. The constitutively expressed EGFP serves as an internal control. The ratio of EGFP to mCherry fluorescence intensity is a quantitative index for the activity of the specific miRNA. With the known miRNA-target pairs as examples, this vector was verified as a good tool to assay miRNA function. MCS1 and MCS2 can be used to easily insert any mRNA target or miRNA, respectively, into the vector. Under the control of three distinct promoters, independent and coordinated expression of the miRNA, indicator and internal control fluorescent proteins occurs from a single construct. In the assay, only one plasmid is transfected into the cell, which reduces the risk of fluctuating gene expression from co-transfections. Compared with traditional luciferase systems, this method does not require rupturing the cells to measure the signals, which makes it useful for monitoring miRNA activity in single living cells. In this study, some miRNA activities were visualized using microscopy to image GFP and mCherry in living cells (see Figure 3). Moreover, because the signal reduction of mCherry is due to miRNA repression of the targets in the 3′-UTR of the mCherry gene, the new vector system might also be used for the functional study of native miRNAs in living cells. Therefore, the new dual-fluorescent protein vector is a simple and reliable system to assay miRNA function.

In our work, miRNAs encoded by the DNA virus SV40 were aligned with the human-derived miRNAs listed in miRBase (http://microrna.sanger.ac.uk/). sv40-miR-S1-5p was found to contain a seed sequence identical to that of the human the derived-miRNA hsa-miR423-5p, suggesting that these two miRNAs from different species might be functional orthologs. Based on our dual-fluorescent protein vector system, we found that sv40-miR-S1-5p and hsa-miR423-5p downregulate the expression of common artificial target mRNAs. Some computationally predicted cellular targets of hsa-miR423-5p, such as DMWD and C20orf27, (http://www.targetscan.org/vert_50/), were also downregulated by both hsa-miR423-5p and sv40-miR-S1-5p. These results demonstrated that SV40-encoded miRNA miR-S1-5p should act as a functional ortholog of the human-derived miRNA miR423-5p. SV40-encoded miR-S1-5p was reported to downregulate the expression of viral T antigen without reducing the yield of infectious virus, thus reducing host cytotoxic T lymphocyte (CTL) susceptibility and local cytokine release. This dispensable down-regulation appears to be very helpful in maintaining the long-term relationship between the virus and the host during latent viral infection or virus-mediated tumorigenesis [7]. Because the cellular miR423-5p may act as a functional ortholog of SV40 miR-S1-5p, it is possible that cellular miR423-5p may also function during latent viral infection or virus-mediated tumorigenesis. DMWD is a widely expressed protein and was suggested to be involved in some of the neuropathological aspects of myotonic dystrophy (DM1) [20]. Little is known about additional functions of DMWD or C20orf27. Nevertheless, the orthologous role of viral miR-S1-5p with cellular miR423-5p also implies that SV40-encoded miRNA not only autoregulates its viral gene expression but also may regulate cellular gene expression, which may play roles in virus-host interactions.

HIV-1-encoded hiv1-miR-N367 was also found by sequence alignment to contain a seed sequence identical to that of the cellular miRNA hsa-miR192. Till now, the function of hiv1-miR-N367 is much less known than some other HIV-1-encoded miRNAs such as the major HIV-1 miRNA encoded by TAR RNA [21], [22]. Based on our dual-fluorescent protein vector system, we found that hiv1-miR-N367 and hsa-miR192 down regulate the expression of common artificial target mRNAs and the predicted cellular target of hsa-miR192, PABPC4. These results demonstrated that these miRNAs should be a pair of functional orthologs. It was reported that the poly(A)-binding protein (PABP) is related to translational repression of host cells during HIV infection [23], [24]. PABPC4, also called inducible PABP (iPABP), was first cloned from activated T cells and shown to be a cellular homolog of PABP [25]. PABPC4 was reported to be expressed at low levels in resting T cells but is drastically induced following activation; thus, PABPC4 might be necessary for regulating the stability of labile mRNA species in activated T cells, such as newly activated lymphokine mRNA [25]. This induction of PABPC4 coincides with the latent life cycle of HIV-1 in resting and activated T cells, thus implying that HIV-1 miR-N367 might repress the activation of lymphokine mRNA expression through targeting PABPC4 to help maintain viral latency. Therefore, the results identifying the orthologous function of HIV-1 miR-N367 with hsa-miR192 imply that HIV-1 may take advantage of endogenous cellular miRNAs to benefit viral latency in resting primary CD4+ T lymphocytes. HIV-1-encoded miRNAs, may also downregulate cellular gene expression, such as PABPC4, to benefit viral latency.

Until now, to our knowledge, the only examples of orthologous relationships between viral and cellular miRNAs were human miR155 and two DNA tumor virus-encoded miRNAs, miR-K12-11 and miR-M4. However, our analysis of currently known viral miRNAs revealed that some other viral miRNAs display seed homology to cellular miRNAs, suggesting that the functional orthologs human miR155 and the two virus-encoded miRNAs are not a unique case. In this study, two pairs of functional orthologs, sv40-miR-S1-5p and hsa-miR423-5p as well as hiv-1-miR-N367 and hsa-miR192, were demonstrated to be potential functional orthologs. Although the significance of these functional orthologs requires additional studies, these results verify that there are functional orthologs besides cellular miR155 and its viral miRNA counterparts. Thus, the findings here expand upon our current knowledge of functional homologies and imply that a more general phenomenon of orthologous relationships between viral and cellular miRNAs exists.

## Materials and Methods

### Plasmid construction

#### pMGhU6

To construct a dual-fluorescent protein reporter, pmCherry was initially constructed based on pEGFP-C1 (Clontech) in which the EGFP-encoding sequence was replaced by the mCherry-encoding sequence using the Nhe I and Bgl II restriction enzymes. Next, a human U6 promoter was PCR amplified from pSIREN-RetroQ (Clontech) with the following primers: 5′-CAACATACGCGTTCGGGCAGGAAGAGGGCCTATTTCC-3′ and 5′-ATGGATACGCGTGCGGCCGCGACTGATATCCCGGTGTTTCGTCCTTTCCACAAGAT-3′. The U6 PCR fragment was subcloned into pmCherry using Mlu I. The resulting plasmid was named pmCherry-U6. EcoR V and Not I sites were introduced during the PCR amplification of the U6 promoter for subsequent cloning steps. An enhanced green fluorescent protein (EGFP) expression cassette containing a cytomegalovirus (CMV) immediate early promoter and enhancer, EGFP-encoding sequence and an SV40 polyadenylation signal was then PCR amplified from position 358-1944 of pAdTrack-CMV (Clontech) by primers: 5′-TACACAATCCACTACGTGCGCGTTAAGATACATTGATGAG-3′ and 5′-TACACAAACCACGTAGTGTAATAGTAATCAATTACGGGGTC-3′. This PCR fragment was digested with Dra III and cloned into the Dra III site of pmCherry-U6. The resulting plasmid was the dual-fluorescent protein reporter vector, named pMGhU6. The construction of pMGhU6 is outlined in Figure 1.

#### pmiR30 and pmiR30:4tar(30)

The miR30 expression vector pmiR30 was constructed by inserting the miR30 precursor (pre-miR30) sequences into the EcoR V and Not I sites of vector pMGhU6. The pre-miR30 sequences were generated by overlap extension PCR with the following two partially complementary oligonucleotides: 5′-CTCGTGATCTGCGACTGTAAACATCCTCGACTGGAAGCTGTGAAGCCACAGATGGGCTTTCAGT-3′ and 5′-ATGTTATCCGCGGCCGCAAAAACTCGTGGATCCGCAGCTGCAAACATCCGACTGAAAGCCCATC-3′.

For the fluorescence reporter assays, the indicator vector pmiR30:4tar(30) was constructed by the stepwise insertion of four tandem copies of miR30 non-fully complementary target sequence (tar(30)) into the 3′-UTR of the mCherry gene in pmiR30 using the Bgl II/Hind III and Hind III/EcoR I restriction sites. The negative control vector pmiR30(mut):4tar(30), which produces no miRNAs, was constructed by deleting the miRNA-encoding region.

#### pmiR-N367 and pmiR-N367:4tar(n)

The miRNA expression vector pmiR-N367 was constructed by inserting the HIV-1 pre-miR-N367 sequences into the EcoR V and Not I sites of pMGhU6. The pre-miR-N367 sequences were generated by overlap extension PCR with the following two partially complementary oligonucleotides: 5′-ATTGGCAGAATTACACACCAGGGCCAGGGATCAGATATCCACTGACCTTTGGATGG-3′ and 5′-ATGTTATCCGCGGCCGCAAAAAACTAGCTTGA AGCACCATCCAAAGGTCAGTGGAT-3′.

The indicator vector pmiR-N367:4tar(n) was made by the stepwise insertion of four tandem copies of miR-N367 non-fully complementary target sequence (tar(n)) into the 3′-UTR of the mCherry gene in pmiR-N367 using the Bgl II/Hind III and Hind III/EcoR I restriction sites. The negative control vector pmiR(mut):4tar(n), which produces no miRNAs, was constructed by deleting the miRNA-encoding region.

#### pmiR-S1-5p, pmiR423-5p and pmiR192

The miRNA expression vector pmiR-S1-5p was constructed by inserting the artificial pre-miR-S1-5p sequences that are based on the miR30 precursor stem-loops into the EcoR V and Not I sites of pMGhU6. The artificial pre-miR-S1-5p sequences were generated by overlap extension PCR with the following two partially complementary oligonucleotides: 5′- TGAGCGCAGGCTCATTTCATGCCCCTCATGGTGAAGCCACAGATGCATGAGGGGCCTG-3′ and 5′-ATTGTTATCGCGGCCGCAAAAAATAGGCAAAGGCTCATTTCAGGCCCCTCATGCATCT-3′.

The miRNA expression vector pmiR423-5p was constructed by inserting the miR30 precursor stem-loops based artificial pre-miR423-5p sequences into the EcoR V and Not I sites of pMGhU6. The artificial pre-miR-S1-5p sequences were generated by overlap extension PCR with the following two partially complementary oligonucleotides: 5′-TGAGCUCAAGTCTCGCTCTCCGCCCCTCATGGTGAAGCCACAGATGCATGAGGGGCAGA-3′ and 5′- ATTGTTATCGCGGCCGCAAAAATAGGCGAAAGTCTCGCTCTCTGCCCCTCATGC-3′.

The miRNA expression vector pmiR192 was constructed by inserting the miR30 precursor stem-loops based artificial pre-miR192 sequences into the EcoR V and Not I sites of pMGhU6. The artificial pre-miR192 sequences were generated by overlap extension PCR with the following two partially complementary oligonucleotides: 5′-TGAGCGAGCTGTCAATTCCTAGGTCAGTGGTGAAGCCACAGATGCACTGACCTATG-3′ and 5′-ATTGTTATCGCGGCCGCAAAAAATAGGCAGGCTGTCAATTCATAGGTCAGTGCATC-3′.

#### pmiR-S1-5p:4tar(s), pmiR423-5p:4tar(s), pmiR-S1-5p:4tar(423-5), and pmiR423-5p:4tar(423-5)

The indicator vectors pmiR-S1-5p:4tar(s) and pmiR423-5p:4tar(s) were constructed by the stepwise insertion of four copies of miR-S1-5p non-fully complementary target sequence (tar(s)) into the 3′-UTR of the mCherry gene in pmiR-S1-5p and pmiR423-5p using the Bgl II/Hind III and Hind III/EcoR I restriction sites, respectively. The negative control vector pmir(mut):4tar(s), which produces no miRNAs, was constructed by deleting the miRNA-encoding region.

The indicator vectors pmiR-S1-5p:4tar(423-5) and pmiR423-5p:4tar(423-5) were constructed by the stepwise insertion of four copies of miR423-5p non-fully complementary target sequence (tar(423-5)) into the 3′-UTR of the mCherry gene in pmiR-S1-5p and pmiR423-5p using the Bgl II/Hind III and Hind III/EcoR I sites, respectively. The negative control vector pmir(mut):4tar(423-5), which produces no miRNAs, was constructed by deleting the miRNA-encoding region.

#### pmiR-S1-5p:C20orf27UTR, pmiR423-5p:C20orf27UTR, pmiR-S1-5p:DMWDUTR, and pmiR423-5p:DMWDUTR

The indicator vectors pmiR-S1-5p:C20orf27UTR and pmiR423-5p:C20orf27UTR were constructed by inserting the full-length 3′-UTR sequence of the human gene C20orf27 into the 3′-UTR of the mCherry gene in pmiR-S1-5p and pmiR423-5p using the Bgl II/EcoR I sites, respectively. The full–length C20orf27 3′-UTR sequence was PCR amplified from human genomic DNA using the following primers: 5′-CCCAGATCTTAAGAGGCCCCGCCTGCCCCGGGCCCCTCAGCCTTA-3′ and 5′-CCCGAATTCCACTGGAAGTTTATTTCTTTAGGGTTCTATCC-3′.

The indicator vectors pmiR-S1-5p:DMWDUTR and pmiR423-5p:DMWDUTR were constructed by insertion of a truncated version of the 3′-UTR sequence of the human gene DMWD (about position 300–800) into the 3′-UTR of the mCherry gene in pmiR–S1–5p and pmiR423–5p using the Bgl II/EcoR I sites, respectively. The truncated version of the DMWD 3′-UTR sequence was PCR amplified from human genomic DNA using the following primers: 5′-CCCAGATCTTAAGAGACTCCCCGCCCCTGCCACAAGAG-3′ and 5′-CCCGAATTCTGATAATTTAAAAAACACCGAGGACTTTG-3′.

The negative control vectors pmir(mut):C20orf27UTR and pmir(mut):DMWDUTR, which produce no relevant miRNAs, were constructed by deleting the pre-miRNA-encoding region. The negative control vectors pmiR-S1-5p:C20orf27UTR(mut), pmiR423-5p:C20orf27UTR(mut), pmiR-S1-5p:DMWDUTR(mut) and pmiR423-5p:DMWDUTR(mut), which contain no relevant miRNA binding sites within the relevant 3′-UTRs, were constructed by deleting the relevant miRNA binding sites using the overlapping PCR technique.

#### pmiR192:4tar(n), pmiR-N367:4tar(192) and pmiR192:4tar(192)

The indicator vector pmiR192:4tar(n) was constructed by the stepwise insertion of four copies of the tar(n) sequence into the 3′-UTR of the mCherry gene in pmiR192 using the Bgl II/Hind III and Hind III/EcoR I sites. The negative control vector pmir(mut):4tar(n), which produces no miRNAs, was constructed previously.

The indicator vectors pmiR-N367:4tar(192) and pmiR192:4tar(192) were constructed by the stepwise insertion of four copies of the non-fully complementary miR192 target sequence (tar(192)) into the 3′-UTR of the mCherry gene in pmiR-N367 and pmiR192 using the Bgl II/Hind III and Hind III/EcoR I, respectively. The negative control vector pmir(mut):4tar(192), which produces no miRNAs, was constructed by deleting the miRNA-encoding region.

#### pmiR-N367:PABPC4UTR and pmiR192:PABPC4UTR

The indicator vectors pmiR-N367:PABPC4UTR and pmiR192:PABPC4UTR were constructed by inserting the full-length 3′-UTR sequence of the human gene PABPC4 into the 3′-UTR of the mCherry gene in pmiR-N367 and pmiR192 using the Bgl II/Sal I sites, respectively. The full-length PABPC4 3′-UTR sequence was PCR amplified from human genomic DNA using the following primers: 5′-CCCAGATCTTAAGATTCAAAAGCCAAATAACCCCTTATGGAATTC-3′ and 5′-CCCGTCGACCAGTTTTATGGAGATTTTTTTTCTTTATTGGGAAACG-3′.

The negative control vector pmir(mut):PABPC4UTR, which produces no relevant miRNAs, was constructed by deleting the pre-miRNA-encoding region. The negative control vectors pmiR-N367:PABPC4UTR(mut) and pmiR192:PABPC4UTR(mut), which contain no relevant miRNA-binding sites within the relevant 3′-UTRs, were constructed by deleting the relevant miRNA-binding sites using the overlapping PCR technique.

All of the plasmids described above were sequenced and verified to be correct The sequences of miRNAs and their targets are shown in Figure 2, 4 and 6The sequences of the pre-miRNAs are listed in Table S1.

### Cell culture and transfection

HeLa cells were purchased from China Center for Type Culture Collection (CCTCC) and cultured in Dulbecco's modified Eagle's medium (DMEM) that was supplemented with 10% fetal bovine serum, 100 U/ml penicillin and 100 U/ml streptomycin at 37°C under a 5% CO_2_ atmosphere. All cell culture reagents were obtained from Gibco-BRL. At 80% confluence, transfections were performed with Lipofectamine 2000 (Invitrogen) according to the manufacturer's instructions.

### Total RNA extraction and Northern blotting

HeLa cells were grown in 100-mm-diameter dishes and transfected individually with DNA or mock transfected. At 48 hours post-transfection, the total RNA that contained miRNA was extracted using the miRNeasy Mini Kit (Qiagen) according to the manufacturer's instructions. Total RNA (five micrograms) was fractionated on a denaturing 12% polyacrylamide gel containing 8 M urea, transferred to a Hybond-N^+^ nylon membrane (Amersham Pharmacia Biotech) by electrophoresis and fixed by ultraviolet cross-linking. The membranes were probed with ^32^P-labeled DNA oligonucleotides that were complementary to the mature microRNAs. Each oligonucleotide probe (10 pmol) was end-labeled with [γ-^32^P] ATP using T4 polynucleotide kinase. Prehybridization of the filters was carried out in ExpressHyb solution (Clontech) at 42°C for 1 hour. Hybridizations were performed in the same fresh solution at 37°C for 2 hours. The labeled probes were heated for 1 min at 95°C before addition to the filters in the hybridization solution. After hybridization, the membranes were washed two times at low stringency in 2× SSC, 0.1% SDS at 35°C for 5 min and one time at high stringency in 0.1× SSC, 0.1% SDS at 35°C for 5 min. Membranes were then exposed to a PhosphorImager screen, and images were analyzed using a Cyclone Storage Phosphor System (PerkinElmer).

The sequences of the probes that were used for Northern blotting are listed in Table S2.

### Fluorescence reporter assay

For the fluorescence reporter assay, transfections were performed in 24-well plates. HeLa cells were harvested at 48 hours post-transfection with individual DNA, resuspended in 100 μL PBS in each well and then transferred to a CulturPlate^TM^-96F (PerkinElmer). The fluorescence intensity was measured using a Synergy HT (Bio-Tek Instruments) with an excitation wavelength of 485±20 nm and an emission wavelength of 528±20 nm for the EGFP channel and an excitation wavelength of 590±20 nm and an emission wavelength of 645±40 nm for the mCherry channel. The ratio of EGFP to mCherry fluorescence intensity was then determined.

### Fluorescence microscopy

HeLa cells were grown in glass-bottom dishes (35-mm-diameter) and transfected with individual DNA. At 48 hours post-transfection, fluorescence images of cells were acquired with a Leica TCS SP2 laser scanning confocal microscope (Leica) that was equipped with a 63×/1.3 NA oil immersion lens using the 488 nm line of an Ar laser (EGFP) and the 543 nm line of an HeNe laser (mCherry). All images captured with each laser line were taken using the same exposure time.

## Supporting Information

Figure S1 **Sequence of MCS1 of vector pMGhU6.**
(TIF)

Figure S2 **Secondary structure models for artificial pre-miRNA based on stem-loops of miR-30 precursor.** A. Secondary structure model for artificial pre-miR-S1-5p based on stem-loops of miR-30 precursor; B. Secondary structure model for artificial pre-miR423-5p based on stem-loops of miR-30 precursor; C. Secondary structure model for artificial pre-miR192 based on stem-loops of miR-30 precursor.
(TIF)

Figure S3 **The 3′ UTR sequences of predicted biological targets and the predicted pairing site of specific miRNAs (marked in red).** A. The 3′UTR sequence of DMWD and the predicted pairing site of hsa-miR423-5p; B. The 3′ UTR sequence of C20orf27 and the predicted pairing site of hsa-miR423-5p; C. The 3′ UTR sequence of PABPC4 and the predicted pairing site of hsa-miR192.
(TIF)

Figure S4 **Relative expression level determination of miR30, miR423-5p and miR192 using real-time quantitative PCR method.**
(TIF)

Figure S5 **Fluorescence reporter assay of different amounts of indicator vectors pMGhU6, pmiR30:4tar(30) and pmiR-N367:4tar(n).** The ratio of EGFP to mCherry fluorescence intensity is shown. **Real-time PCR assay.** HeLa cells were grown in 100-mm-diameter dishes and transfected individually with DNA or mock transfected. At 48 hours post-transfection, the total RNA that contained miRNA was extracted using the miRNeasy Mini Kit (Qiagen) according to the manufacturer's instructions. cDNA synthesis was carried out with the Superscript III cDNA synthesis kit (Invitrogen) using 1°μg of total RNA as the template and specific reverse primers under 16°C, 30°min, 42°C, 30°min and 85°C, 5°min of reverse transcription. The specific reverse primer for U6 was 5′-CGCTTCACGAATTTGCGTGTCAT-3′, and the reverse primers for miR30, miR423-5p and miR192 were 5′- GTCGTATCCAGTGCGTGTCGTGGAGTCGGCAATTGCACTGGATACGACGCTGCAA-3′, 5′-GTCGTATCCAGTGCGTGTCGTGGAGTCGGCAATTGCACTGGATACGACAAAGTCT-3′ and 5′-GTCGTATCCAGTGCGTGTCGTGGAGTCGGCAATTGCACTGGATACGACGGCTGTC-3′ respectively. The resulting cDNA was amplified by PCR using miRNA specific primers with SYBR Premix Ex Taq (Takara). Primers for U6 were 5′-GCTTCGGCAGCACATATACTAAAAT-3′ and 5′-CGCTTCACGAATTTGCGTGTCAT-3′; primers for miR30 were 5′-CAGTGCGTGTCGTGGAGT-3′ and 5′-GCCCCTTTCAGTCGGATGT-3′; primers for miR423-5p were 5′-CAGTGCGTGTCGTGGAGT-3′ and 5′-GCCCTGAGGGGCAGAGAGC-3′; primers for miR423-5p were 5′-CAGTGCGTGTCGTGGAGT-3′ and 5′- GCCCCTGACCTATGAATTG-3′. PCR parameters were as follows: 95°C for 30 s, followed by 40 cycles of 95°C for 5 s, 60°C for 34 s. At the end of the PCR cycles, melting curve analysis was performed. The expression of miR30, miR423-5p and miR192 was compared to mock transfected sample using 2^–ΔΔCT^ method.
(TIF)

Table S1 **Sequences of pre-miRNAs.** * Artificial pre-miRNAs which were designed being based on stem-loops of miR-30 precursor (ref 9). Mature sequences of each miRNAs are marked in red.
(DOC)

Table S2 **Sequences of probes used for northern blot.**
(DOC)
