# Supplementary figures and images for: Functionally Orthologous Viral and Cellular MicroRNAs Studied by a Novel Dual-Fluorescent Reporter System
Source: PLoS One. 2012 Apr 27;7(4):e36157. doi: 10.1371/journal.pone.0036157 (PMC3338597; doi:10.1371/journal.pone.0036157)

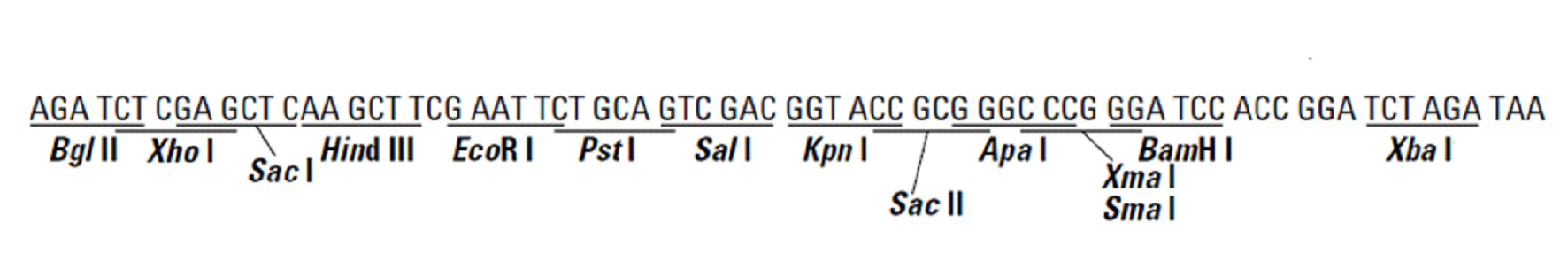

Supplement: Figure S1 — Sequence of MCS1 of vector pMGhU6. (TIF) [file pone.0036157.s001.tif]

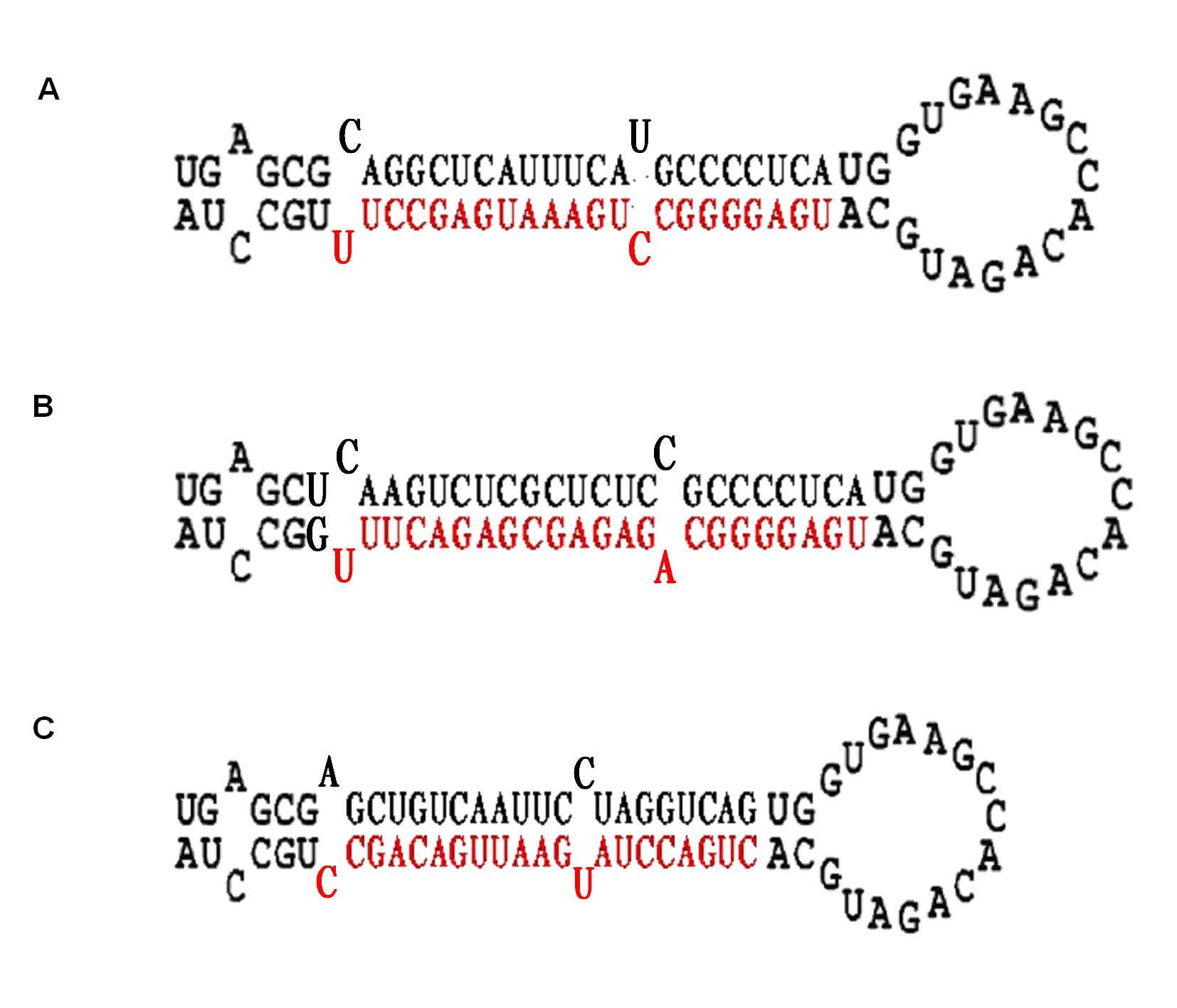

Supplement: Figure S2 — Secondary structure models for artificial pre-miRNA based on stem-loops of miR-30 precursor. A. Secondary structure model for artificial pre-miR-S1-5p based on stem-loops of miR-30 precursor; B. Secondary structure model for artificial pre-miR423-5p based on stem-loops of miR-30 precursor; C. Secondary structure model for artificial pre-miR192 based on stem-loops of miR-30 precursor. (TIF) [file pone.0036157.s002.tif]

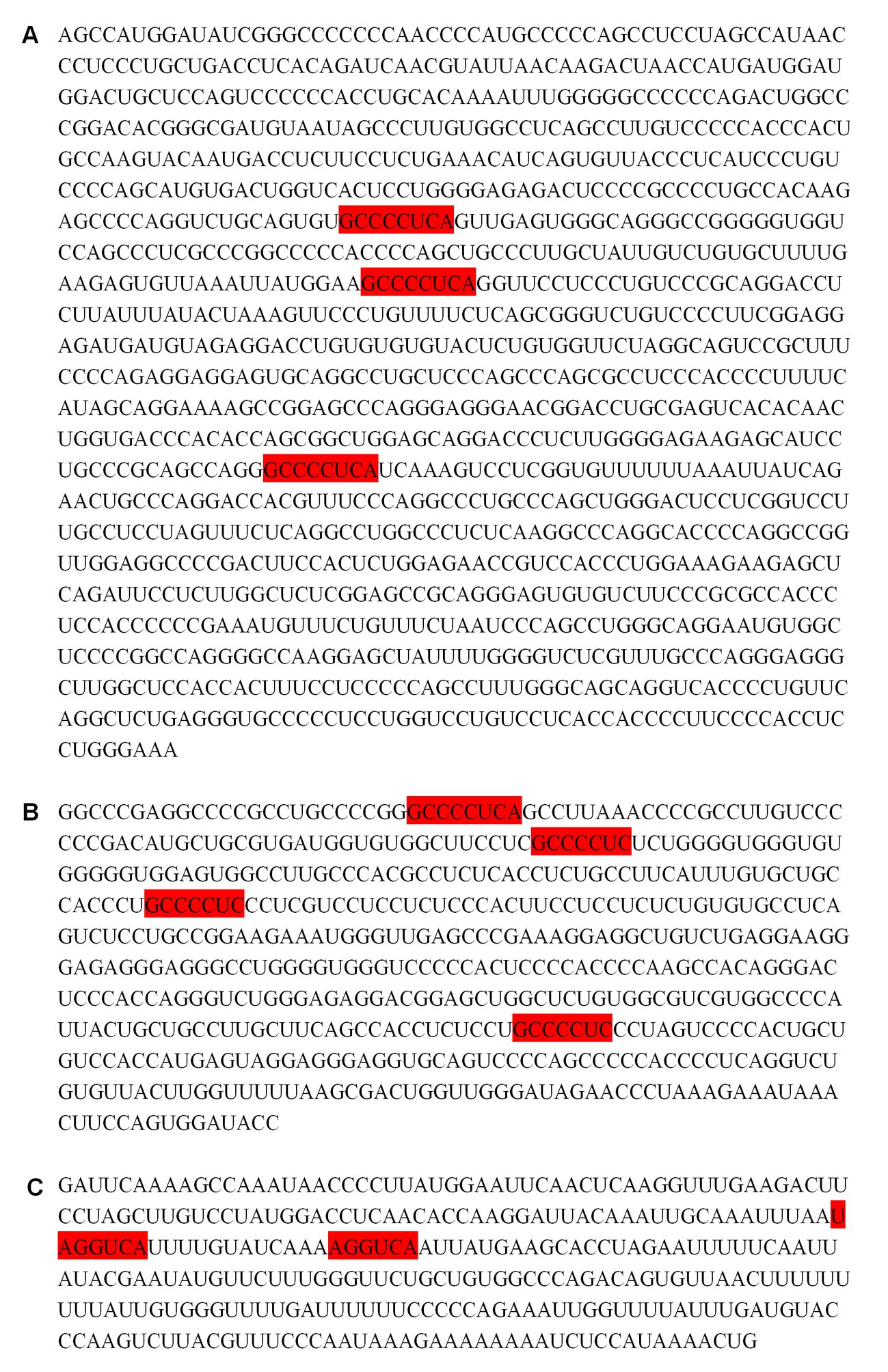

Supplement: Figure S3 — The 3′ UTR sequences of predicted biological targets and the predicted pairing site of specific miRNAs (marked in red). A. The 3′UTR sequence of DMWD and the predicted pairing site of hsa-miR423-5p; B. The 3′ UTR sequence of C20orf27 and the predicted pairing site of hsa-miR423-5p; C. The 3′ UTR sequence of PABPC4 and the predicted pairing site of hsa-miR192. (TIF) [file pone.0036157.s003.tif]

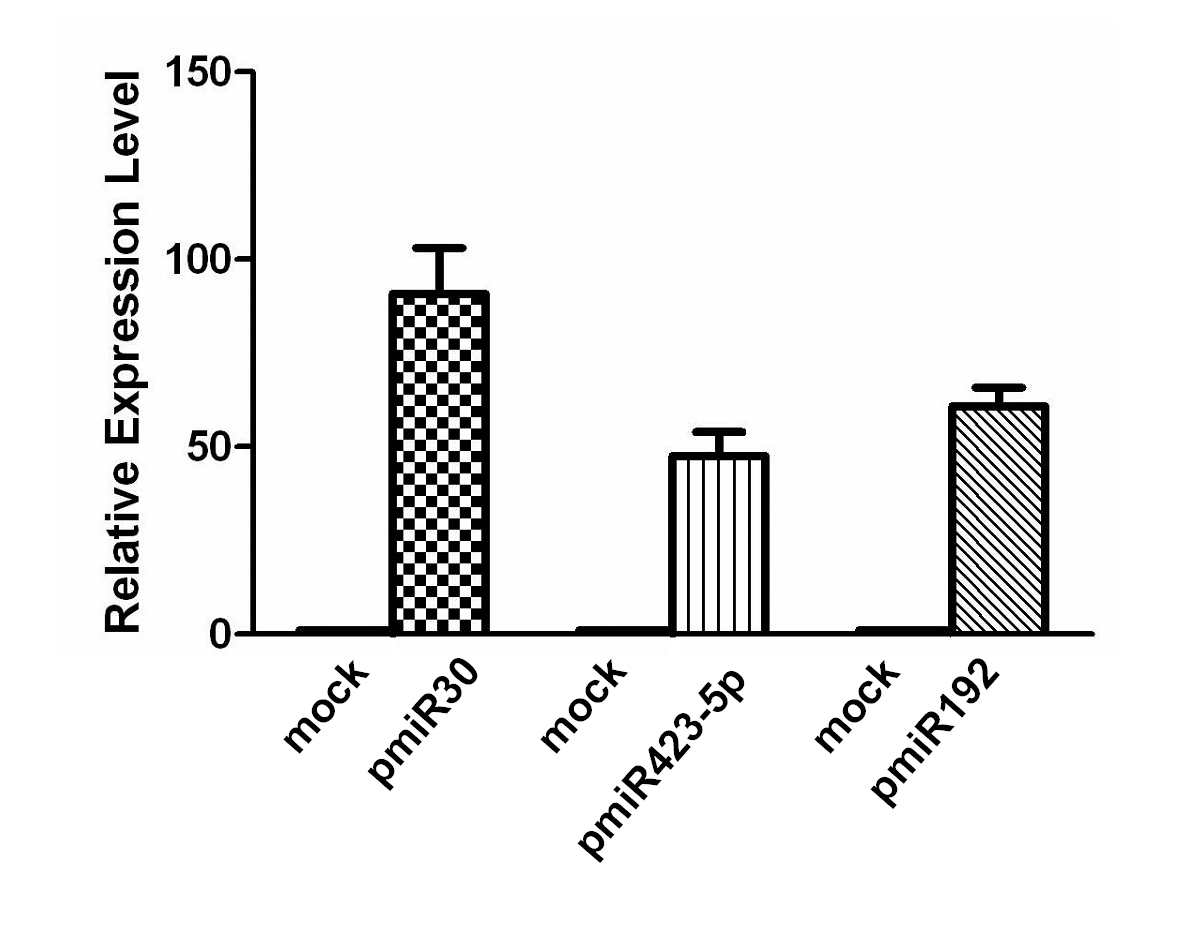

Supplement: Figure S4 — Relative expression level determination of miR30, miR423-5p and miR192 using real-time quantitative PCR method. (TIF) [file pone.0036157.s004.tif]

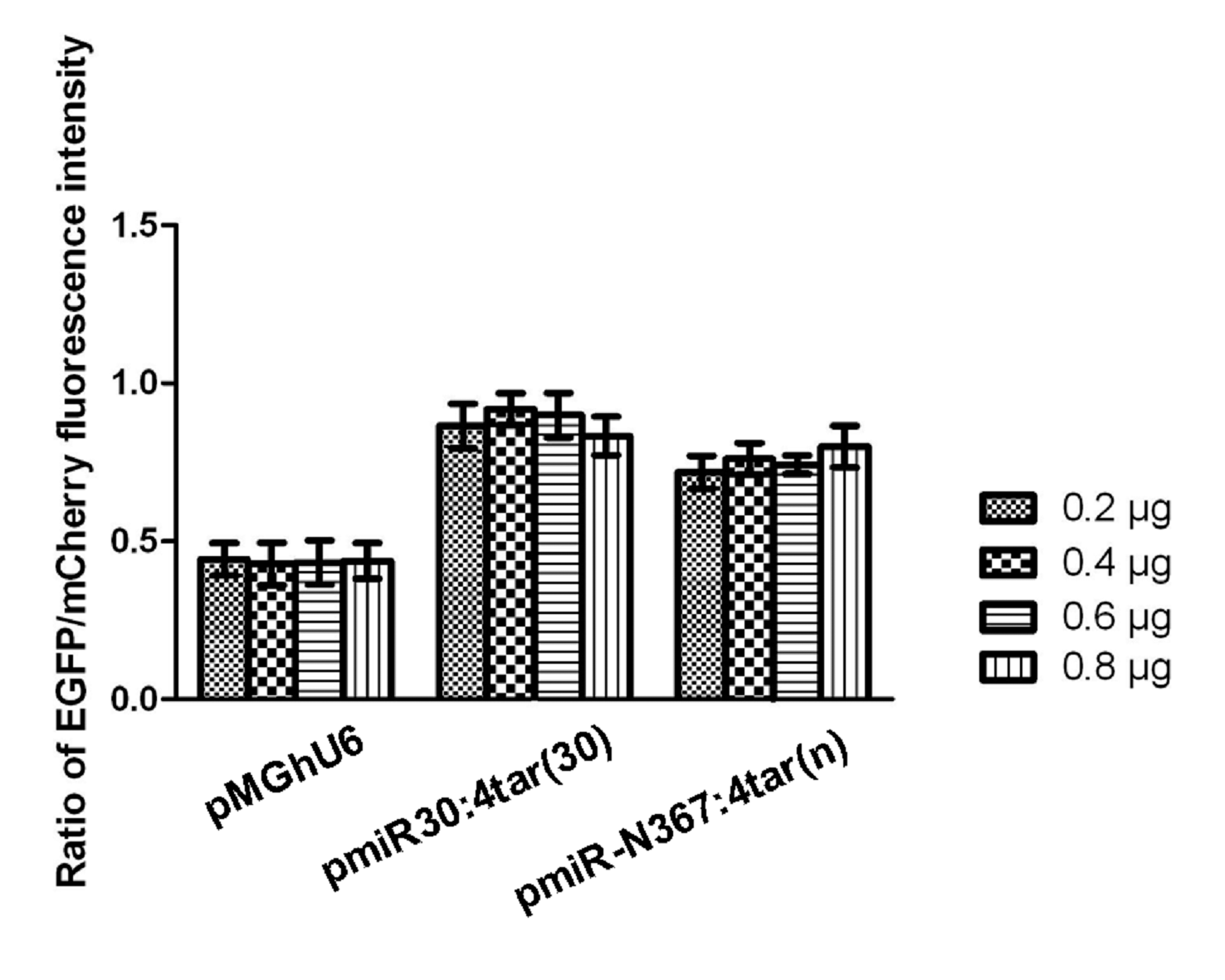

Supplement: Figure S5 — Fluorescence reporter assay of different amounts of indicator vectors pMGhU6, pmiR30:4tar(30) and pmiR-N367:4tar(n). The ratio of EGFP to mCherry fluorescence intensity is shown. Real-time PCR assay. HeLa cells were grown in 100-mm-diameter dishes and transfected individually with DNA or mock transfected. At 48 hours post-transfection, the total RNA that contained miRNA was extracted using the miRNeasy Mini Kit (Qiagen) according to the manufacturer's instructions. cDNA synthesis was carried out with the Superscript III cDNA synthesis kit (Invitrogen) using 1°μg of total RNA as the template and specific reverse primers under 16°C, 30°min, 42°C, 30°min and 85°C, 5°min of reverse transcription. The specific reverse primer for U6 was 5′-CGCTTCACGAATTTGCGTGTCAT-3′, and the reverse primers for miR30, miR423-5p and miR192 were 5′- GTCGTATCCAGTGCGTGTCGTGGAGTCGGCAATTGCACTGGATACGACGCTGCAA-3′, 5′-GTCGTATCCAGTGCGTGTCGTGGAGTCGGCAATTGCACTGGATACGACAAAGTCT-3′ and 5′-GTCGTATCCAGTGCGTGTCGTGGAGTCGGCAATTGCACTGGATACGACGGCTGTC-3′ respectively. The resulting cDNA was amplified by PCR using miRNA specific primers with SYBR Premix Ex Taq (Takara). Primers for U6 were 5′-GCTTCGGCAGCACATATACTAAAAT-3′ and 5′-CGCTTCACGAATTTGCGTGTCAT-3′; primers for miR30 were 5′-CAGTGCGTGTCGTGGAGT-3′ and 5′-GCCCCTTTCAGTCGGATGT-3′; primers for miR423-5p were 5′-CAGTGCGTGTCGTGGAGT-3′ and 5′-GCCCTGAGGGGCAGAGAGC-3′; primers for miR423-5p were 5′-CAGTGCGTGTCGTGGAGT-3′ and 5′- GCCCCTGACCTATGAATTG-3′. PCR parameters were as follows: 95°C for 30 s, followed by 40 cycles of 95°C for 5 s, 60°C for 34 s. At the end of the PCR cycles, melting curve analysis was performed. The expression of miR30, miR423-5p and miR192 was compared to mock transfected sample using 2–ΔΔCT method. (TIF) [file pone.0036157.s005.tif]
