# Supplementary material for: Functionally Orthologous Viral and Cellular MicroRNAs Studied by a Novel Dual-Fluorescent Reporter System
Source: PLoS One. 2012 Apr 27;7(4):e36157. doi: 10.1371/journal.pone.0036157 (PMC3338597; doi:10.1371/journal.pone.0036157)
Supplement: Table S1 — Sequences of pre-miRNAs. * Artificial pre-miRNAs which were designed being based on stem-loops of miR-30 precursor (ref 9). Mature sequences of each miRNAs are marked in red. (DOC) [file pone.0036157.s006.doc]

| pre-miRNAs | Sequence |
| --- | --- |
| miR30 | AUCUGCGACUGUAAACAUCCUCGACUGGAAGCUGUGAAGCCACAGAUGGGCUUUCAGUCGGAUGUUUGCAGCUGCGGAU |
| miR-N367 | UUGGCAGAAUUACACACCAGGGCCAGGGAUCAGAUAUCCACUGACCUUUGGAUGGUGCUUCAAGCUAGUA |
| miR-S1-5p* | UGAGCGCAGGCUCAUUUCAUGCCCCUCAUGGUGAAGCCACAGAUGCAUGAGGGGCCUGAAAUGAGCCUUUGCCUA |
| miR423-5p* | UGAGCUCAAGUCUCGCUCUCCGCCCCUCAUGGUGAAGCCACAGAUGCAUGAGGGGCAGAGAGCGAGACUUUGGCCUA |
| miR192* | UGAGCGAGCUGUCAAUUCCUAGGUCAGUGGUGAAGCCACAGAUGCACUGACCUAUGAAUUGACAGCCUGCCUA |
