# Supplementary material for: Functionally Orthologous Viral and Cellular MicroRNAs Studied by a Novel Dual-Fluorescent Reporter System
Source: PLoS One. 2012 Apr 27;7(4):e36157. doi: 10.1371/journal.pone.0036157 (PMC3338597; doi:10.1371/journal.pone.0036157)
Supplement: Table S2 — Sequences of probes used for northern blot. (DOC) [file pone.0036157.s007.doc]

| probe | Sequence |
| --- | --- |
| tRNA^Val^ | CTAAGTGTAAGTTGGGTGCTTTGTGTTAAGCTACACTCTG |
| hsa-miR30 | GCTGCAAACATCCGACTGAAAG |
| hiv1-miR-N367 | TTGAAGCACCATCCAAAGGTCAGT |
| sv40-miR-S1-5p | AAGGCTCATTTCAGGCCCCTCA |
| hsa-miR423-5p | AAAGTCTCGCTCTCTGCCCCTCA |
| hsa-miR192 | GGCTGTCAATTCATAGGTCAG |
